# Peptide receptor radionuclide therapy controls inappropriate calcitriol secretion in a pancreatic neuro-endocrine tumor: a case report

**DOI:** 10.1186/s12876-020-01470-1

**Published:** 2020-10-02

**Authors:** Maarten Haemels, Thierry Delaunoit, Koen Van Laere, Eric Van Cutsem, Chris Verslype, Christophe M. Deroose

**Affiliations:** 1 Nuclear Medicine, University Hospitals Leuven, Herestraat 49, 3000 Leuven, Belgium; 2 Medical Oncology, Hospital of Jolimont, Rue Ferrer 159, 7100 La Louvière, Belgium; 3 Department of Imaging and Pathology, Nuclear Medicine and Molecular Imaging, KU Leuven, Herestraat 49, 3000 Leuven, Belgium; 4 Digestive Oncology, University Hospitals Leuven, Herestraat 49, 3000 Leuven, Belgium; 5 Department of Oncology, KU Leuven, Herestraat 49, 3000 Leuven, Belgium

**Keywords:** ^177^Lu-DOTATATE, PRRT, Calcitriol, Neuroendocrine tumor, Pancreas, Vitamin D, Hypercalcemia

## Abstract

**Background:**

Hypercalcemia of malignancy is not uncommon in patients with advanced stage cancer. In rare cases the cause of the hypercalcemia is excessive production of calcitriol, the active form of vitamin D. Although inappropriate tumoral secretion of calcitriol is typically associated with lymphomas and some ovarian germ cell tumors, we present a case of calcitriol overproduction-induced hypercalcemia due to a pancreatic neuroendocrine tumor. The high expression of somatostatin receptors on this neuroendocrine neoplasm opened up the opportunity to treat the patient with radiolabelled somatostatin analogs, which successfully controlled the refractory hypercalcaemia and calcitriol levels. This case documents a rare finding of refractory hypercalcaemia of underlying malignancy due to a calcitriol-producing pancreatic neuroendocrine tumor, responding to peptide receptor radionuclide therapy (PRRT).

**Case presentation:**

A 57 years-old patient presented with back pain, general discomfort, polydipsia, polyuria, fatigue and recent weight loss of 10 kg. Clinical examination was normal and there was no relevant medical history. Biochemical evaluation showed hypercalcemia with markedly increased calcitriol levels. CT-thorax-abdomen and ultrasound guided biopsy revealed a pancreatic neuroendocrine tumor with multifocal liver metastases, suggesting that excessive overproduction of calcitriol by this neuroendocrine tumor was the cause of the refractory hypercalcemia. The patient was eligible for PRRT. Four cycles of ^177^Lu-DOTATATE PRRT resulted in a morphological response and a normalization of serum calcium levels, confirming the hypothesis of a calcitriol producing pancreatic neuroendocrine tumor. Progression of liver metastases warranted further therapy and temozolomide-capecitabine was started with morphological and biochemical (serum calcium, calcitriol) stabilization.

**Conclusion:**

Although up to 30–40% of gastroenteropancreatic neuroendocrine tumors are known to be functional (i.e. producing symptoms associated with the predominant hormone/peptide secreted), calcitriol secreting pancreatic neuroendocrine tumors are very rare. However, treatment with PRRT resulted in normalization of calcium and calcitriol levels, strongly supporting the hypothesis of a calcitriol-producing pancreatic neuroendocrine tumor.

## Background

Hypercalcemia without elevated parathyroid hormone (PTH) level is most frequently due to an underlying neoplasm [1, 2]. This so called hypercalcemia of malignancy (HCM) is a common finding, affecting up to 44% of cancer patients [3, 4] and is most often associated with advanced tumor stages [4]. Up to 80% of HCM are caused by systemic secretion of PTH-related Peptide (PTHrP) [1, 3] which mimics the function of PTH: increasing kidney calcium reabsorption, stimulating the maturation of osteoclast precursors and increasing bone resorption [5]. Extensive osteolysis accounts for about 20% of the cases of HCM [1, 6]. In rare cases (< 1%) the cause of the hypercalcemia is an excessive production of calcitriol (1,25-dihydroxyvitamin D), due to an ectopic overexpression of 1-alpha-hydroxylase [1, 7, 8]. The latter is common in lymphomas and in some ovarian germ cell tumors [1]. However, we report it in a grade 2 pancreatic neuroendocrine tumor (pNET).

The high incidence of somatostatin receptors (SSR) on gastroenteropancreatic neuroendocrine tumors (GEP-NETs) provides imaging and therapeutic options. Somatostatin receptors overexpression is the basis of pharmacological treatment with non-radioactive somatostatin analogs (SSAs) (eg. Octreotide) [9]. SSAs can also be labelled with radionuclides [10–13]. Depending on the specific decay characteristics of the attached radionuclide, the compound can be used for diagnostic molecular imaging (eg. ^68^Ga-DOTATATE) or targeted radionuclide therapy (eg. ^177^Lu-DOTATATE). In case of inoperable/metastatic well-differentiated GEP-NETs, this so-called Peptide Receptor Radionuclide Therapy (PRRT) is an established treatment modality [14] whose role has been proven by the excellent results obtained in the randomized controlled NETTER-1 trial [15]. Furthermore, up to 30–40% of the GEP-NETs are known to be functional (i.e. causing symptoms mediated by excessive hormone/peptide secretion) [16], and evidence is accumulating that PRRT is valuable in controlling these functional neuroendocrine tumor syndromes [17–22]. This case documents a rare finding of refractory hypercalcaemia of underlying malignancy due to a calcitriol-producing pancreatic neuroendocrine tumor, responding to peptide receptor radionuclide therapy (PRRT).

## Case presentation

A 57 years-old male patient presented with back pain, general discomfort, polydipsia, polyuria, fatigue and recent weight loss of 10 kg. Clinical examination was normal and there was no relevant medical history. Biochemical evaluation showed hypercalcemia (2,85 mmol/L; normal: 2.15–2.55 mmol/L) with slightly lowered PTH levels (14,2 ng/L; normal 14,9–56,9 ng/L) excluding hyperparathyroidism. CT-thorax-abdomen and ultrasound guided biopsy revealed a pNET with multifocal liver metastases as well as some small bone lesions. The Ki-67 index was 15 to 20% compatible with a grade 2 tumor. PTHrP was normal and although our patient had some osteodense skeletal metastases, these bone lesions alone could not explain his marked hypercalcemia. However, markedly increased calcitriol levels up to 134.3 ng/L (normal: 20.0–80.0 ng/L) were detected. We hypothesized that overproduction of this active form of vitamin D by the pNET was the cause of the HCM.

**Fig. 1:**
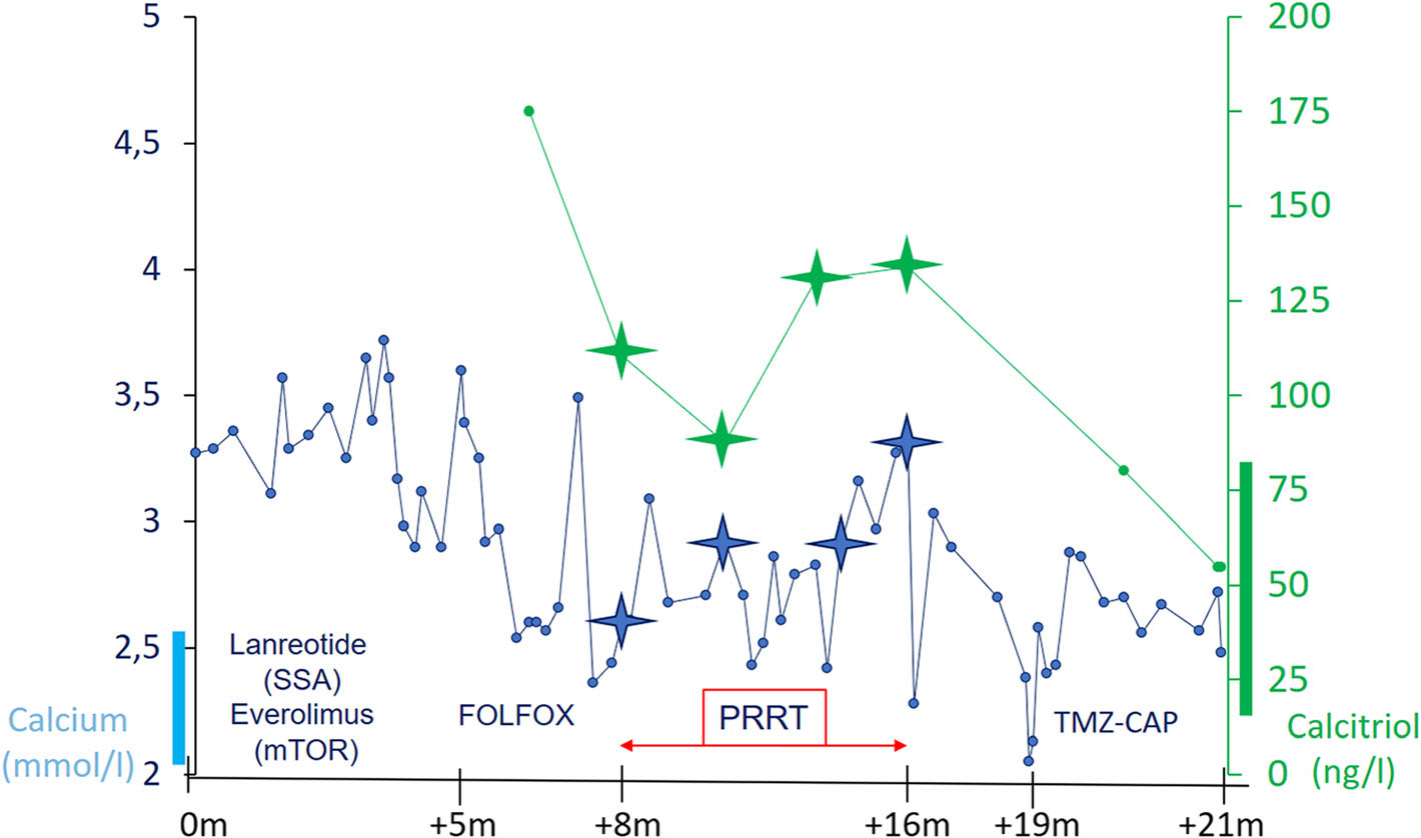
Evolution of calcium and calcitriol during therapy. Normal range of calcium (left) and calcitriol (right) are displayed by a colour bar on the vertical axis. The star symbols represent the values at each cycle of PRRT

**Fig. 2:**
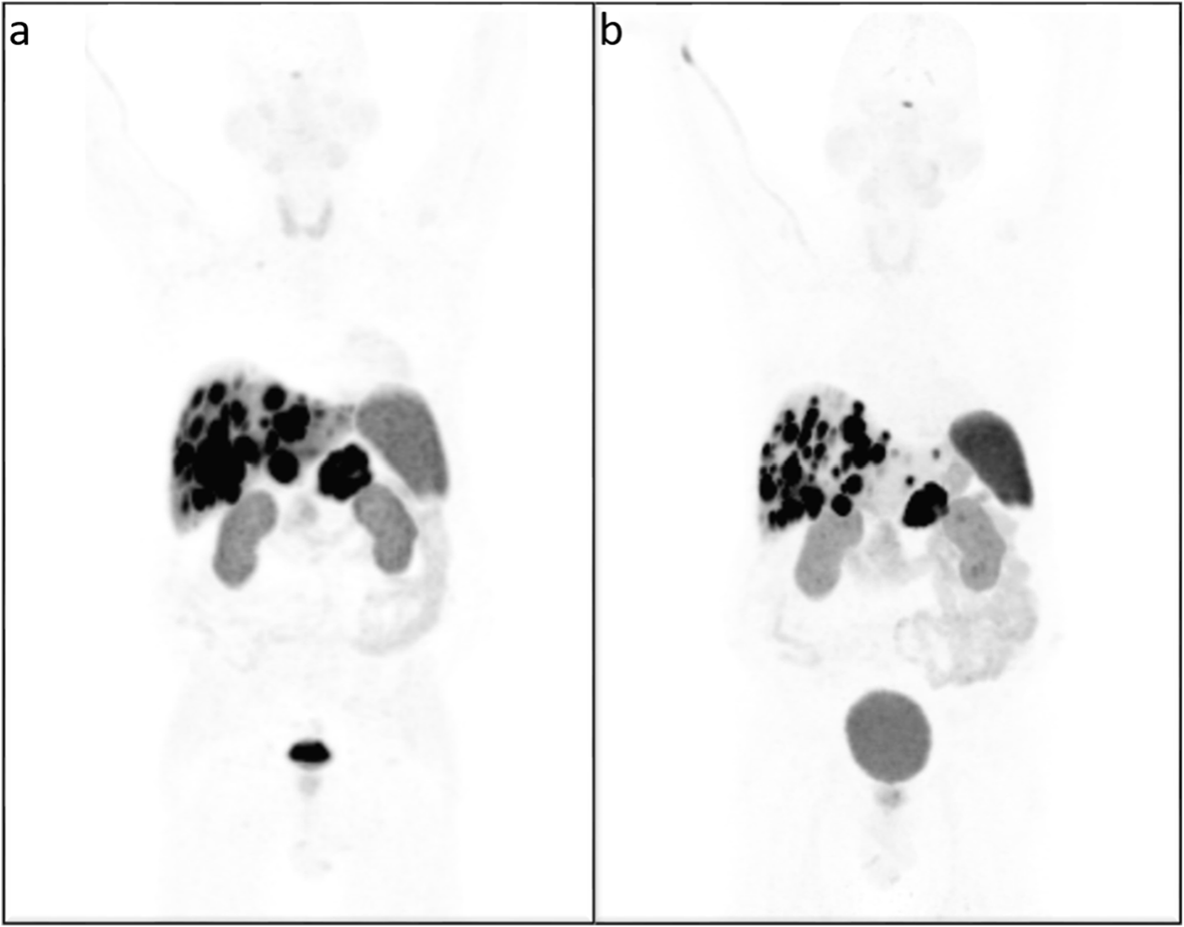
^68^Ga-DOTATATE PET-CT MIP image before PRRT (**a**) compared to the post-PRRT image (**b**)

**Fig. 3:**
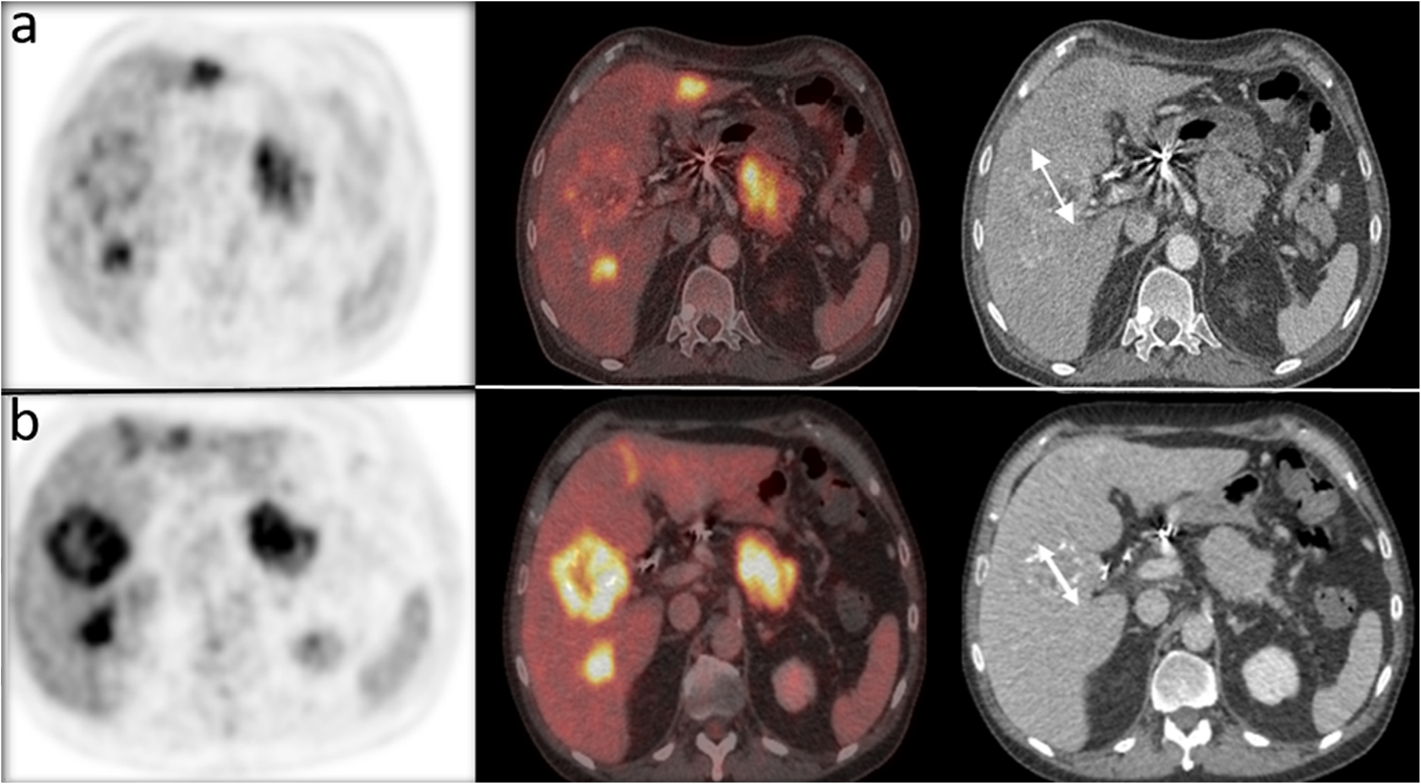
^18^F-FDG-PET/CT transverse images before PRRT (row **a**) compared to post-PRRT images (row **b**). Some liver lesions showed an increase in ^18^F-FDG uptake compared with baseline (left an middle images), although there was a morphologic therapy response on the CT images (right images) with new calcifications and a decrease in diameter (white arrow)

Initial treatment with lanreotide, a non-radioactive SSA, and everolimus, an inhibitor of mammalian target of rapamycin (mTOR), resulted in morphologically stable disease, but there was no effect on the hypercalcemia nor on the associated symptoms. The patient was evaluated for treatment with PRRT. In the meantime, therapy with FOLFOX chemotherapy was started. ^68^Ga-DOTATATE scan revealed intense SSR expression in the pancreatic lesion as well as strong uptake in the liver metastases and the skeletal metastases. All malignant lesions had an uptake intensity above the spleen (Krenning score grade 4) [23]. ^18^F-FDG-PET/CT showed strong hypermetabolism in some of the liver metastases (metabolic grade 3) [24]. There were no ^18^F-FDG + / SSR - mismatched lesions. Evaluation of the renal function showed no contraindication for therapy. Four cycles of PRRT with ^177^Lu-DOTATATE were given, with a treatment interval of 8 weeks up to a cumulative activity of 29.6 GBq. Three months after the final cycle, the initially refractory serum calcium levels had normalized and the associated symptoms disappeared (Fig. 1), confirming the hypothesis of a calcitriol secreting pNET. Although there was a clear morphologic response (Figs. 2 and 3), some liver lesions showed an increase in ^18^F-FDG uptake compared with baseline (Fig. 3). Because of these signs of metabolic progression, the patient was started on temozolomide-capecitabine, which resulted in continued morphological disease stabilization as well as continued normal serum calcium and calcitriol.

## Discussion and conclusion

Hypercalcemia without elevated PTH level is most frequently due to HCM. However, the normal PTHrP values and the absence of osteolytic bone metastases, ruled out PTHrP production and skeletal osteolysis respectively. Instead, the strongly elevated calcitriol levels suggested a third, more rare cause of HCM: calcitriol overproduction-induced hypercalcemia due to a pNET. To the best of our knowledge, only 2 other cases have described calcitriol-producing pNETs [25, 26], both in a metastatic setting. In the case report of Zhu et al. [25] biochemical disease stabilization was obtained using a combination of temozolomide-capecitabine as well as a 4-weekly regimen of octreotide. The case report of Van Lierop et al. [26] documented disease control after hemihepatectomy. In our case, initial therapy with lanreotide and everolimus had no effect on the hypercalcemia nor on the associated symptoms, so we opted to treat the patient with PRRT as PRRT can reduce hormonal secretion by functioning NETs.

^68^Ga-DOTATATE scan revealed intense SSR expression in the pancreatic lesion, the liver metastases and the rare skeletal metastases. Strong hypermetabolism in some of the liver metastases, suggested increased aggressiveness in those lesions [27]. However, no mismatched (^18^F-FDG + / SSR -) lesions were detected, so there was no significant dedifferentiation which would be a contra-indication for treatment with PRRT [28]. Since PRRT response is observed months after first administration (between 3 to 12 months or even later), the fluctuating calcium levels during PRRT treatment were probably caused by a combination of the symptomatic treatment with hydration and forced diuresis and the PRRT effect However, a marked decrease in serum calcium as well as a morphological therapy response were observed three months after completion of PRRT, in line with the PRRT response kinetics. The biochemical and morphologic alterations after PRRT make it highly likely that calcitriol decreased concomitantly, although it was not measured between the end of PRRT and the start of chemotherapy and was only confirmed to be normal after additional chemotherapy. Although no ex-vivo biochemical proof of overexpression of 1-alpha-hydroxylase was available, our findings strongly support the hypothesis of a calcitriol-producing pNET and demonstrate for the first time the potential role of PRRT in controlling inappropriate tumoral secretion of calcitriol. This is in line with other data documenting successful PRRT treatment of different kinds of secreting NETs, proving PRRT to be a valuable asset in controlling functional neuroendocrine tumor syndromes.

Neuroendocrine neoplasms are known to be functional in up to 30–40% of the cases, but calcitriol secretion by a pNET has only rarely been described. However, treatment with PRRT resulted in normalization of calcium and calcitriol levels, strongly supporting the hypothesis of a calcitriol-producing pancreatic neuroendocrine tumor.

## Data Availability

Not applicable.

## Abbreviations

PTH: Parathyroid hormone
HCM: Hypercalcemia of malignancy
PTHrP: PTH-related Peptide
pNET: Pancreatic neuroendocrine tumor
SSR: Somatostatin receptor
GEP-NETs: Gastroenteropancreatic neuroendocrine tumors
SSA: Somatostatin analog
DOTATATAE: DOTA-(Tyr3)-octreotate
PRRT: Peptide receptor radionuclide therapy
CT: Computed tomography
mTOR: Inhibitor of mammalian target of rapamycin
FOLFOX: Oxaliplatin 5-fluorouracil leucovorin
18F-FDG: 18F-Fluorodeoxyglucose
PET: Positron emission tomography
